# Antitumor activity of mixed heat shock protein/peptide vaccine and cyclophosphamide plus interleukin-12 in mice sarcoma

**DOI:** 10.1186/1756-9966-30-24

**Published:** 2011-02-26

**Authors:** Quan-Yi Guo, Mei Yuan, Jiang Peng, Xue-Mei Cui, Ge Song, Xiang Sui, Shi-Bi Lu

**Affiliations:** 1 Institute of Orthopedic Research, General Hospital of the People's Liberation Army, Beijing 100853, China

## Abstract

**Background:**

The immune factors heat shock protein (HSP)/peptides (HSP/Ps) can induce both adaptive and innate immune responses. Treatment with HSP/Ps in cancer cell-bearing mice and cancer patients revealed antitumor immune activity. We aimed to develop immunotherapy strategies by vaccination with a mixture of HSP/Ps (mHSP/Ps, HSP60, HSP70, Gp96 and HSP110) enhanced with cyclophosphamide (CY) and interleukin-12 (IL-12).

**Methods:**

We extracted mHSP/Ps from the mouse sarcoma cell line S180 using chromatography. The identity of proteins in this mHSP/Ps was assayed using SDS-PAGE and Western blot analysis with antibodies specific to various HSPs. BALB/C mice bearing S180 cells were vaccinated with mHSP/Ps ×3, then were injected intraperitoneally with low-dose CY and subcutaneously with IL-12, 100 μg/day, ×5. After vaccination, T lymphocytes in the peripheral blood were analyzed using FACScan and Cytotoxicity (CTL) was analyzed using lactate dehydrogenase assay. ELISPOT assay was used to evaluate interferon γ (IFN-γ), and immune cell infiltration in tumors was examined in the sections of tumor specimen.

**Results:**

In mice vaccinated with enhanced vaccine (mHSP/Ps and CY plus IL-12), 80% showed tumor regression and long-term survival, and tumor growth inhibition rate was 82.3% (30 days), all controls died within 40 days. After vaccination, lymphocytes and polymorphonuclear leukocytes infiltrated into the tumors of treated animals, but no leukocytes infiltrated into the tumors of control mice. The proportions of natural killer cells, CD8+, and interferon-γ-secreting cells were all increased in the immune group, and tumor-specific cytotoxic T lymphocyte activity was increased.

**Conclusions:**

In this mice tumor model, vaccination with mHSP/Ps combined with low-dose CY plus IL-12 induced an immunologic response and a marked antitumor response to autologous tumors. The regimen may be a promising therapeutic agent against tumors.

## Introduction

Some of the most abundant proteins in the cell belong to the well-conserved family of proteins known as heat shock proteins (HSPs), or glucose-regulated proteins (GRPs). HSPs are present in all living cells; they can exist in an unbound state or a state bound to specific client proteins. HSPs function as molecular chaperones in numerous processes, such as protein folding, assembly and transport, peptide trafficking, and antigen processing under physiologic and stress conditions [1,2]. Levels of HSPs are elevated in many cancers [3,4]. One of the first identified HSP subtypes, Gp96, can reject tumors [5]. HSP as a natural adjuvant can elicit in cancer patients a specific and active autoimmune response to a tumor [6]. During tumor formation, HSPs increase and bind to exposed hydrophobic tumor polypeptides. HSP-chaperoned peptides enter antigen-presenting cells through specific receptors and prime T cells by increasing major histocompatibility complex (MHC) class I and II-mediated antigen presentation [7-9]. The relevance of the peptides associated with HSPs for inducing specific immune responses is demonstrated by numerous studies, and GRP96, HSP70, HSP110 and GRP170 purified from diverse tumors and functioning as tumor vaccines have shown to cause tumor regression in animal models [10-13]. The factor is successful in CD8^+ ^T cell-dependent tumor clearance. The immune recognition does not come from HSPs themselves but from binding to peptides [14]. Some HSPs, such as HSP60 and HSP70, augment natural killer (NK) cell activity, which can also elicit innate immune responses [15,16].

As an alternative to selecting a single antigen for tumor vaccine development, random mutations in cancer cells generate antigens unique to an individual. Purification of chaperone HSP from a cancer is believed to co-purify an antigenic peptide "fingerprint" of the cell of origin [17]. Thus, a vaccine comprising HSP/peptide (HSP/P) complexes derived from a tumor, which would include a full repertoire of patient-specific tumor antigens, obviates the need to identify cytotoxic T-lymphocyte (CTL) epitopes from individual cancers. This advantage extends the use of chaperone-based immunotherapy to cancers for which specific tumor antigens have not yet been characterized [18].

After an extensive study, HSPs were found to augment tumor antigen presentation and NK cell activity leading to tumor lysis. Autologous patient-specific tumor vaccines have been generated by purifying HSP-antigen complexes from tumor specimens and are currently being evaluated in clinical trials. Preliminary clinical trials with Gp96 used as a personalized vaccine for immunotherapy in melanoma, renal, colon, ovarian cancer and non-Hodgkin lymphoma have reported results [19-23]. HSP70 as a vaccine for leukemia was studied in a clinical trial [24]. Although various immunotherapeutic approaches have been examined for the treatment of cancer, no such therapy has entered into the clinical standard of care, and the therapeutic effects was not satisfactory. Several challenges still need to be overcome.

Until now, all clinical trials have used the single subtype of HSPs, Gp96 or HSP70, whereas in a few animal tumor models, the combination of Gp96 and HSP70 has been shown to possess antitumor activity superior to the that of each type alone [25]. These results suggest that the mixture of several HSP subtypes may be more effective in a broad range of tumor models. We used the mixture of HSP/Ps (mHSP/Ps) that include HSP60, HSP70, HSP110 and GRP96 as a vaccine and found an effective prophylactic antitumor effect of the mHSP/Ps in a mouse sarcoma model [26,27]. The effect protected against tumor challenge in 50% of immunized mice, but this strategy for the therapeutic treatment in already established tumors were not satisfactory, so enhancing the therapeutic immunity is needed.

Using cytokines to enhance immune reactivity has been reported both in experimental and clinical trials [28]. Interleukin 12 (IL-12) is still the most important single cytokine in inducing antitumor immunity. In experimental tumor models, recombinant IL-12 has demonstrated marked antitumor effects through mechanisms of both innate and adaptive immunity [29,30]. The most unique antitumor activity of IL-12 is its ability to eradicate established tumors [31,32]. However, the significant antitumor activity of IL-12 in these models requires the presence of pre-existing immunity in tumor-bearing hosts [33]. Thus, further improvement of IL-12-based immunotherapy also depends on the combination of vaccine-based modalities to establish pre-existing immunity in tumor-bearing hosts.

When patients are diagnosed with cancer, by definition, the tumor has "escaped" the immune system, having passed the phases of "elimination" and "equilibrium." The generation of immune response against these antigens is likely unproductive in the late stage because of multiple immune tolerance mechanisms such as Treg infiltration in the tumor bed, general immune suppression from immunosuppressive cytokines producing by tumor cells, and downregulation of MHC class I molecules on the tumor cells. Also, myeloid-derived suppressor cells (MDSCs) and tumor-associated macrophages (TAMs) create an immunosuppressive environment that leads to suppression of T-cell responses [34,35]. Thus, multiple immunological "brakes" need to be lifted to augment a productive immune response. Combined immunotherapeutic modalities need to be seriously considered. The use of combination therapy with more than one agent or modality is needed. To overcome the multiple immune tolerance mechanisms, combinations of anticancer drugs and immunotherapy have been shown to enhance tumor immunotherapy [36,37]. Treating mice with low-dose cyclophosphamide (CY) decreased the number of Tregs and enhanced the immunostimulatory and antitumor effects [38-40].

To improve the efficacy of tumor immunotherapy, we used the mHSP/P vaccine as an agent to induce pre-existing immunity in a tumor-bearing mouse host, and combined with CY plus IL-12 to eradicate established large tumors in a therapeutic antitumor mouse model.

## Methods

### Animals and Cell Lines

6-8 weeks-old female BALB/C mice were obtained from the Military Medical Academy of China (Beijing) and bred in the General Hospital of the People's Liberation Army. The institutional animal care and use committee approved the study protocols. The ascetic mouse S180 sarcoma cell line was obtained from the Military Medical Academy of China. The cell line was maintained by serial passages in the BALB/C mouse peritoneal cavity.

#### Reagents

Anti-HSP60, anti-HSP70, anti-HSP110 and anti-Gp96/94 antibodies were obtained from Santa Cruz Biotechnology (Santa Cruz, CA, USA). Sephacryl S-200HR, concanavaline A (ConA) and adenosine 5'-diphosphate (ADP) affinity column were obtained from Pharmacia (US). Recombinant murine IL-12 was provided by Dr. K. Tsung at the Stanford School of Medicine. CY was obtained from Heng Ray Pharmaceutical Co. (Jiangsu, China).

#### HSP/P vaccine

mHSP/Ps were isolated from fresh, solid S180 subcutaneous tumors implanted in BALB/C mice. Tumor tissue was homogenized by the use of a homogenizer at 4°C in buffer (30 mM NaHCO_3_, pH 7.1) with freshly added protease inhibitor phenyl-methylsulfonyl fluoride (0.5 mM). The homogenate was centrifuged at 10,000 g for 30 min at 4°C and the supernatant was then centrifuged at 100,000 g at 4°C for 2 h. The resulting supernatant was dialyzed against 20 mM Tris-HCl and 150 mM NaCl, pH 7.2, and then was applied to Sephacryl S-200HR. Bovine serum albumin was used as a molecular indicator in a pilot experiment to map the range of eluted fractions. The tumor supernatant protein was eluted with the same sample loading buffer. The collected fractions of eluted protein underwent SDS-PAGE. The fractions of #3 to #6 contained proteins of about 40-200 kDa. The combination of these 4 fractions was used as the mHSP/Ps vaccine. The identity of proteins in this combination was assayed using SDS-PAGE and Western blot analysis with antibodies specific to various HSPs.

#### In vivo antitumor experiments

To evaluate the antitumor activity of the mHSP/Ps preparation, mice were divided into 6 groups for treatment (n = 10 mice each): 1) normal saline control, 2) mHSP/Ps, 3) CY plus IL-12, 4) mHSP/Ps plus IL-12, 5) mHSP/Ps plus CY, 6) mHSP/Ps plus Cy plus IL-12.

All mice were subcutaneously injected in the back with 5 × 10^4 ^S180 cells. One day later, groups Groups 2, 4, 5, and 6 mice were vaccinated 3 times at 7-day intervals with 20 μg of mHSP/Ps. Groups 5 and 6 received 2 mg of CY intraperitoneally 1 day after the last vaccination. Groups 4 and 6 mice were subcutaneously injected with IL-12, 100 ng/day, for 5 days, 3 days after a CY injection. Group 3 mice received CY plus IL-12 at the same time as Group 6, but the treatment started on day 16.

The antitumor effects were evaluated by tumor volume, tumor growth inhibition rates, metastasis rate and overall survival time. Tumor volume was determined by the measurement of the shortest (A) and longest diameter (B) using a caliper once every 3 days. The volume (V) was calculated by the formula V = (A^2^B/2). Curative survival was considered to occur when the tumor did not regrow or disappeared after more than 3 months. Lungs, liver and brains of dead mice were removed and fixed in formalin, embedded in paraffin, and sectioned at 5 μm. Hematoxylin & eosin (H&E) stained samples were examined under a light microscope (Olympus).

#### Analysis of immune response

Treatment of mice for analysis of immune responses was the same as that for immunotherapy. Three days after the combined therapy of mHSP/Ps and CY plus IL-12, all mice were killed, and blood and spleen samples were collected. Mice from various control groups were killed at the same time.

##### Assay for subgroup of T cells

T lymphocytes in the peripheral blood were analyzed using FACScan (Becton Dickinson); cell staining involved a use of FITC- or phycoerythin-conjugated goat antibodies against mouse CD4+, CD8+ and NK cells (Serotect, UK).

##### Cytotoxicity assays (CTL)

Lactate dehydrogenase assay was used to assess *in vitro *tumor-specific CTL response to immunization with mHSP/Ps or mHSP/Ps and CY plus IL-12. Three days after the final IL-12 administration, splenocytes were isolated by Ficoll-Paque density centrifugation and were used as effector cells after restimulation with ConA and mHSP/Ps *in vitro *for 4 days. S180 as target cells were seeded in 96-well plates. The lymphocytes were serially diluted and plated in 96-well plates in triplicate with varying E:T ratios of 40:1, 20:1 and 5:1. Wells containing only target cells or only lymphocytes with culture medium or 0.5% Triton X-100 served as spontaneous or maximal release controls. After 4-h incubation at 37°C and 5% CO_2_, 150-ul supernatant was analyzed in a Well scan at OD 490 nm (BioRad); the percentage of specific lysis was calculated as follows:

% specific lysis = 100 × (experimental release - spontaneous release)/(maximum release - spontaneous release).

##### ELISPOT assay for evaluating interferon γ (IFN-γ)

Splenocytes were isolated by Ficoll-Paque density centrifugation. 2 × 10^5 ^cells were incubated with ConA (8 μg/ml) or additionally restimulated with mHSP/Ps (10 μg/ml) for 5 days in 96-well ELISPOT plates coated with antibody to bind murine IFN-γ. The assays followed the kit manufacturer's instructions (U-CyTech B.V. Holland).

##### Immune cell infiltration in tumors

Tumor tissue was removed after mice were killed, fixed in formalin, embedded in paraffin, and sectioned at 5 μm. H&E-stained tissues were examined under a light microscope.

### Statistical analysis

All experiments were performed in triplicate, and the data were presented as mean± SD. Statistical analysis involved a use of SPSS 13.0 (SPSS Inst., Chicago, IL). Data were shown as means ± SD. A two-tailed paired *t *test with Welch correction was used for comparison of IFN-γ levels of the experimental and control groups. A P < 0.05 was considered statistically significant.

## Results

### Preparation of mHSP/Ps

**Figure 1:**
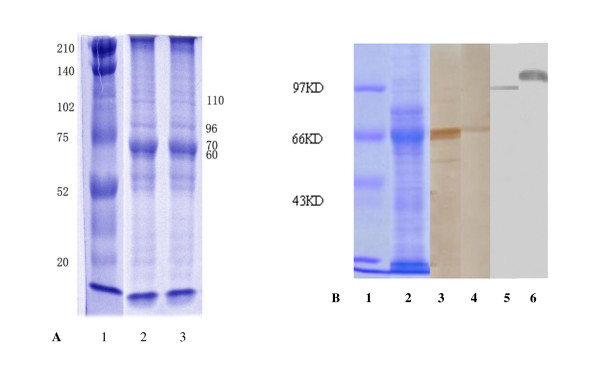
**SDS-PAGE and western blot analysis of mixed HSP/Ps from S180 sarcoma**. A. SDS-PAGE of mHSP/P from S180; Lane1, molecular standard, Line2,3 collection of F3-F6 from Sephacryl S-200HR. There were many protein bands other than MW60, 70, 96 and110. B. Western blot: Lane 1, SDS-PAGE, molecular standard. 2, SDS-PAGE, collection of F3-F6, Line3 analysis with antibodies against HSP60, Line4 analysis with antibodies against HSP70, Line5 analysis with antibodies against Gp96, and Line6 analysis with antibodies against HSP110. Identified The mixture included HSP60, HSP70, Gp96 and HSP110.

The combination of 4 protein fractions was eluted from S180 tumor cells. The presence of the various HSPs -- HSP60, HSP70, Gp96 and HSP110 -- in the crude preparation was identified by SDS-PAGE and Western blot analysis (Figure 1). As indicated in SDS-PAGE, there were many bands for proteins other than HSPs in the sample, and components of HSP60, HSP70, Gp96 and HSP110 were identified by Western blot, with their purity of 90% in total proteins.

### Therapeutic antitumor effects of mHSP/Ps and CY plus IL-12 treatment in mouse sarcoma tumor model

**Figure 2:**
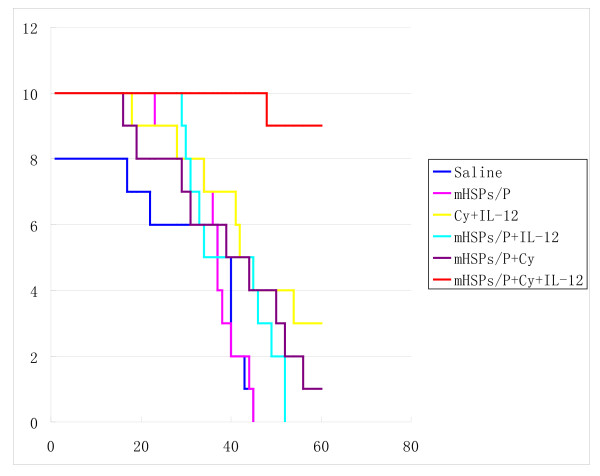
**Effect of various mHSP/P vaccinations on the survival of S180 tumor-bearing mice**. * The number of mouse in each group is 10.

All 10 mice treated with saline alone died within 40 days because of tumor burden. Some of these mice had tumor metastases in the lung before death. Vaccination with mHSP/Ps alone and mHSP/Ps plus IL-12 (starting on day 19) also had no antitumor effects. In mice vaccinated with mHSP/Ps plus CY (day 16), 10% showed eradicated tumors. In mice vaccinated with CY plus IL-12 (starting on day 16), 30% showed eradicated tumors. In comparison, in mice vaccinated with mHSP/Ps followed by Cy plus IL-12 (starting on day 16), 80% showed eradicated tumors (Figure 2). The mean survival time, except long-term survival, for groups was as follows: saline control, 35.5 days; mHSP/Ps, 32.4 days; mHSP/Ps plus IL-12, 40.1 days; mHSP/Ps plus CY, 37.3 days; CY plus IL-1, 37.4 days; and mHSP/Ps plus CY plus IL-12:,48 days.

**Figure 3:**
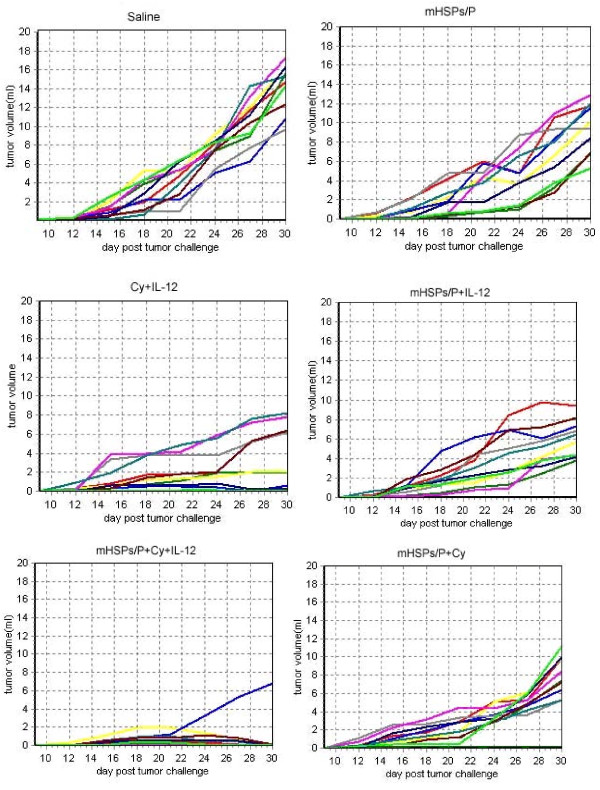
**Tumor growth curve of S180 tumor in BALB/C mice after various treatments**.

The tumor growth curve of S180 tumors in BALB/C mice after vaccination with mHSP/Ps plus CY plus IL-12 was less steep than that for all control groups (Figure 3), so tumor progression was inhibited substantially.

To determine whether this antitumor activity induced long-term immunity against tumors, we challenged mice that survived with 5 × 10^4 ^S180 cells 15 months after the first challenge with the same cell line. No tumors developed in any mice, which indicated that long-term immunological memory against the S180 tumor was associated with tumor eradication by our immunotherapy.

#### mHSP/Ps and mHSP/Ps plus CY plus IL-12 induce immune reaction

##### Change of immune cell population with various vaccinations

In naïve mice, the mean proportion of CD8+ cells in total mononuclear cells was 5.89 ± 0.36%. At the late stage of tumor-bearing (day 26), the proportion of CD8+ T cells was suppressed to 1.26%. Treatment with mHSP/Ps increased the proportion of CD8+ T cells to 9.1 5% at about the same time of tumor establishment (day 26), With mHSP/Ps plus CY plus IL-12 treatment, the CD8+ population was higher (9.21 ± 1.45%) than that in mHSP/P-treated mice and untreated tumor-bearing mice. Similar to the proportion of CD8+ T cells, that of CD4+ T cells was suppressed in late-stage tumor-bearing mice. Treatment with mHSP/Ps plus CY plus IL-12 increased the ratio of CD4+ T cells. In mice treated with normal saline, the mean NK cell in total mononuclear cells was 1.70% ± 0.32%. Again, in tumor-bearing mice, the ratio of NK cells was suppressed to 0.19%. This ratio was increased to 4.98% with mHSP/Ps alone and was even greater with mHSP/Ps plus CY plus IL-12 (5.72%).

##### Number of INF-γ-secreting cells was elevated with mHSP/Ps and CY plus IL-12 vaccination

**Figure 4:**
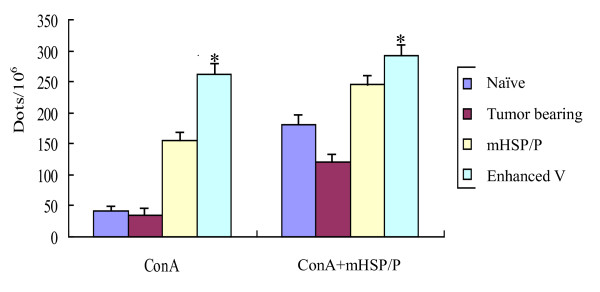
**mHSP/P+Cy+IL12 vaccination elicits IFN-γ by ELISPOT assay ConA: stimulate lymphocyte proliferation *in vitro *with ConA**. ConA+mHSP/P: stimulate lymphocyte proliferation *in vitro *with ConA and mHSP/P. IFN-γ elicited by mHSP/P+Cy+IL12 vaccination is significantly higher than tumor bearing mice and naïve mice, *P < 0.05.

To determine whether vaccination with mHSP/Ps results in increased number of antigen-specific Th1 cells and IFN-γ-producing NK cells, the number of IFN-γ-secreting splenocytes was determined by an *in vitro *assay of IFN-gamma ELISPOT. The frequency of IFN-γ-producing splenocytes increased with ConA alone or ConA plus mHSP/Ps *in vitro *(Figure 4). Under both stimulation conditions, splenocytes from mice treated with both mHSP/Ps alone and mHSP/Ps plus CY plus IL-12 showed an increased number of IFN-gamma-producing cells, with the later treatment giving the higher number. The number of IFN-γ elicited by mHSP/P+Cy+IL12 vaccination was significantly higher than that of tumor bearing mice and naïve mice, P < 0.05.

##### CTLs generated by mHSP/Ps plus CY plus IL12 are capable of killing target cells

**Figure 5:**
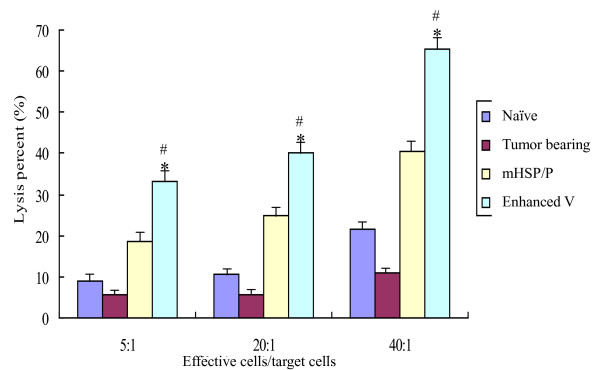
**mHSP/P+Cy+IL12 vaccination elicits a tumor-specific CTL response**. The cytolysis percent of mHSP/P+Cy+IL12 vaccine is significantly higher than mHSP/P vaccine and naïve mice *P < 0.05, and tumor bearing mice, #P < 0.01.

To assess the functional effector properties of CTLs generated by mHSP/Ps plus CY plus IL-12, we performed *in vitro *cytotoxicity assays of lymphocytes isolated from mice treated with mHSP/Ps plus CY plus IL-12. The cytolytic activity of effector cells was measured by lactate dehydrogenase assay. Target cells (S180) pulsed with effector splenocyte cells from mice treated with mHSP/Ps were killed to some extent by CTLs, an amount higher than in those pulsed with splenocytes from naïve mice or tumor-bearing mice not treated with mHSP/Ps (Figure 5). The cytolysis percentage of mHSP/P+Cy+IL12 vaccine was significantly higher than that of mHSP/Ps vaccine and naïve mice, P < 0.05, and that of tumor bearing mice, P < 0.01. In addition, the proportion of lysis of lymphocytes to rabbit liver cancer cells vx2 was very low, 4% in E/T = 5 and 10% in E/T = 20.

##### Lymphocytes and leukocytes were recruited to tumor lesions

**Figure 6:**
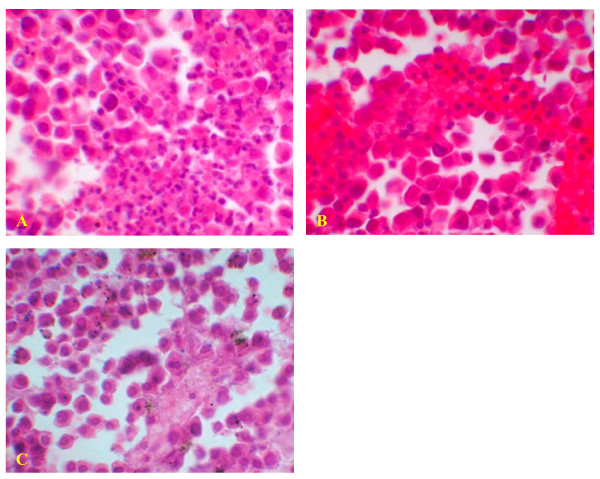
**Lymphocytes infiltration in tumor of mHSP/P immunized mice**. A leukocytes infiltration into tumor lesion after mHSP/P immunization, X40. B lymphocytes in blood vessels after mHSP/P immunization, X40. C No lymphocytes infiltration in tumor lesion after NS treatment, X40. Which revolved preimmunization after mHSP/P immunization.

In histological examination of tumor lesions of immunized mice, leukocytes were found to have infiltrated tumor lesions since numerous lymphocytes were collected in blood vessels and near blood vessel walls, whereas no leukocytes were found to have infiltrated tumors of mice without vaccine (Figure 6). This result showed that pre-immunization was induced after mHSP/Ps immunization.

## Discussion

Vaccination with HSP/Ps is personalized, delivering tumor antigen as a fingerprint genome. The vaccine is polyvalence. Here we developed a vaccine with a mixture of HSP/Ps which, in addition to HSP70 or Gp96, also included HSp60 and HSP110. The antitumor effects of this mHSP/Ps vaccine were more potent than those of HSP70 or HSP60 alone and of tumor lysates used as vaccine in prophylactic immunization, Table 1. [25]. When using this mHSP/P vaccine in mice after tumor transplantation (therapeutic immunization), the antitumor action was not effective, as we showed in this study. The efficacy of therapeutic immunization was effective only in the combination therapy that used immunotherapeutic mHSP/Ps combined with CY and IL-12.

**Table 1:** Comparison of antitumor effects of various HSPs

	Untreated	mHSP/p	HSP70	HSP60	tumor lysate
No. of animals tested	10	10	10	10	10
Complete regression, no. (%)	0	4 (40%)	3 (33.3%)	1 (10%)	2 (20%)
Tumor growth inhibition rate (%)		82.3	62.3	42.6	66.2

For specific immunotherapy, the identical MHC genetic molecules are important, We had no information about the MHC genetic molecules of S180 or MCA-207 when we selected the mouse sarcoma cell lines S180 and MCA-207 as models. However, from reported experimental information and our experiments, we knew that the S180 sarcoma cell lines can grow both in BALB/C and C57 mice, as in our control group, in which all the S180 tumors grew and were not rejected. This finding suggests S180 and BALB/C mice have the matched MHC locus even in allogenic transplantation. The MCA-207 only grew in C57 mice but was rejected in BALB/C mice, and this result suggests that the MHC of MCA-207 matched only with the MHC of C57 mice; therefore, in our animal models, the allogenic immune rejection did not occur, and the results of mHSP/P antitumor effects were not related to unmatched MHC.

To identify the specificity of mHSP/P vaccine, we compared the cytolysis ratio of mHSP/Ps isolated from liver and muscle of naïve mice *in vitro *and saw no cytolytic effect against S180 sarcoma. The cytolysis ratio was lower than 1%. Also, we compared the mHSP/p of S180 against rabbit liver cancer cell line vx2, and the cytolysis effect was lower than 10%, [data not shown]. In addition, we found that the mice vaccinated with mHSP/P of MCA207 were protected only against MCA207 but not S180 *in vivo*. Thus, the mHSP/P-induced immune reaction may be autologous tumor-specific, like individual vaccines.

IL-12 is highly effective against established immunogenic tumors. In our study, the combination of IL-12 and Cy eradicated tumors in 30% of mice, and in IL-12-treated mice, all tumor mass necrosis and an ulcer formed before tumor eradication, suggesting the anti-angiogenesis activity of IL-12 was involved [41], When we combined mHSP/Ps with CY and IL-12 to enhance the immunization efficacy, the antitumor efficacy enhanced. However, with mHSP/Ps and CY alone or with mHSP/Ps and IL-12 alone, the antitumor efficacy was not improved. Our results suggested that one potential mechanism of mHSP/Ps and CY plus IL-12 in augmenting therapeutic immunotherapy strategies was that mHSP/P immunization activated the antitumor immunization, and at the same time, also induced the T-cell tolerance directed toward tumor-associated antigens and limited the repertoire of functional tumor-reactive T cells. Therefore, the ability of vaccines to elicit effective antitumor immunity was impaired. CY has immunomodulatory effects, and low-dose CY (20 mg/kg) was found to selectively deplete CD4+CD25+ T cells (Treg) and impede the tolerance [42]. CY can preconditioning enhance the CD8+ T-cell response to peptide vaccination, thus leading to enhanced antitumor effects against pre-existing tumors [43]. Cy markedly enhanced the magnitude of secondary but not primary CTL response induced by vaccines and synergized with vaccine in therapy but not in prophylaxis tumor models [44].

With our enhanced vaccine, IFN-γ secretion was significantly increased. In addition, CD8+ and NK cells were triggered to release IFN-γ and mediate cytotoxic activity. The increased IFN-γ secretion may also be due to the combined effects of HSP60 in mHSP/P and IL-12. Hsp60-inducing IFN-γ depends strictly on the ability of the macrophages to produce IL-12 [45].

Activation and expansion of tumor-specific T cells by HSP/Ps were identified [46]. Our study showed that mHSP/Ps purified from S180 sarcoma cells activated tumor antigen-specific T cells *in vitro*, and the induction of tumor-specific CTLs with enhanced vaccine was stronger than that with mHSP/Ps alone, possibly because of the combined effect of HSP60 and IL-12. HSP60 induces a strong non-specific immune reaction, but when it meets IL-12, it can activate cytotoxic T cells. HSP60 can mediate the activation of cytotoxic T cells, which depends on production of IL-12 [47].

Our data showed that inflammatory cells infiltrated tumors with mHSP/P vaccination and that a preexisting antitumor immune response was elicited, which was required for an effective IL-12 response for tumor rejection.

## Conclusions

To enhance the current immunotherapeutic efficacy, novel strategies designed in the laboratory and proven in preclinical animal tumor models are now entering the clinic trials [48,49]. These novel strategies involved breaking tolerance to tumor self-antigens by inhibiting regulatory T cells, boosting T-cell co-stimulation and using combinations of recombinant cytokines and other defined molecules with "immuno-enhancing" activities. Our immunization protocol of a combination immunotherapeutic regimen of vaccination with mHSP/Ps followed by low-dose CY plus IL-12 resulted in enhanced immunologic antitumor activity that was better than that of either treatment alone.

## Competing interests

The authors declare that they have no competing interests.

## Authors' contributions

Q-YG The design of the study. MY Conceived and the design of the study, drafted the manuscript. JP Carried out the animal study and performed the statistical analysis. X-MC Preparation the HSP/P vaccine, carried out the immunoassays. GS Carried out the immunoassays. XS Carried out the animal study and the immunoassays. S-BL Conceived of the study. All authors read and approved the final manuscript.
